# Blue Fluorescent cGMP Sensor for Multiparameter Fluorescence Imaging

**DOI:** 10.1371/journal.pone.0009164

**Published:** 2010-02-11

**Authors:** Yusuke Niino, Kohji Hotta, Kotaro Oka

**Affiliations:** Center for Biosciences and Informatics, School of Fundamental Science and Technology, Keio University, Kohoku-ku, Yokohama, Japan; University of Southampton, United Kingdom

## Abstract

Cyclic GMP (cGMP) regulates many physiological processes by cooperating with the other signaling molecules such as cyclic AMP (cAMP) and Ca^2+^. Genetically encoded sensors for cGMP have been developed based on fluorescence resonance energy transfer (FRET) between fluorescent proteins. However, to analyze the dynamic relationship among these second messengers, combined use of existing sensors in a single cell is inadequate because of the significant spectral overlaps. A single wavelength indicator is an effective alternative to avoid this problem, but color variants of a single fluorescent protein-based biosensor are limited. In this study, to construct a new color fluorescent sensor, we converted the FRET-based sensor into a single wavelength indicator using a dark FRET acceptor. We developed a blue fluorescent cGMP biosensor, which is spectrally compatible with a FRET-based cAMP sensor using cyan and yellow fluorescent proteins (CFP/YFP). We cotransfected them and loaded a red fluorescent probe for Ca^2+^ into cells, and accomplished triple-parameter fluorescence imaging of these cyclic nucleotides and Ca^2+^, confirming the applicability of this combination to individually monitor their dynamics in a single cell. This blue fluorescent sensor and the approach using this FRET pair would be useful for multiparameter fluorescence imaging to understand complex signal transduction networks.

## Introduction

cGMP is an important second messenger, which is especially involved in cardiovascular and nervous systems, and regulates cellular functions by cooperating with the other signaling molecules such as cAMP and Ca^2+^ [1]–[3]. To visualize the spatiotemporal dynamics of signal transduction, many biosensors including that for cGMP have been developed based on FRET between CFP and YFP derived from the *Aequorea victoria* green fluorescent protein (GFP) [4], [5]. Recently, some approaches have been reported for combined use of two FRET-based sensors in a single cell [6]–[10]. These approaches for dual FRET imaging are useful to analyze the dynamic relationship between two signaling events. In the previous study, we constructed a FRET-based cGMP sensor using variants of Sapphire and *Discosoma* sp. red fluorescent protein (RFP) for combined use with a cAMP sensor using CFP/YFP [10]. Four-color imaging and subsequent linear unmixing enabled to distinguish the fluorescent proteins, and simultaneous imaging of intracellular cAMP and cGMP in a single cell was accomplished. However, for an advance toward the imaging of three cellular parameters such as these two cyclic nucleotides and Ca^2+^, further addition of a fluorescent biosensor together with two FRET-based sensors would complicate the imaging experiments.

A single wavelength indicator is an important alternative to provide a simple means for multicolor imaging, and use of a circularly permuted variant of *Aequorea* GFP (cpGFP) is an effective method currently available to create a single fluorescent protein-based biosensor [5], [11], [12]. However, its color variants previously reported have been limited to cyan, green and yellow. Thus, existing cpGFP-based sensors are inadequate for combination with the FRET-based sensor using the most commonly employed CFP/YFP pair. In addition, cpGFP-based sensors often exhibit low pH stability [5]. On the other hand, an approach to use a dark YFP as a FRET acceptor for GFP has been recently reported [13], [14]. It leads to an exclusive detection of the emission originating from the donor, and was applied for improving the sensitivity of fluorescence lifetime imaging microscopy (FLIM). We conceived that this approach would be useful to convert the FRET-based sensor, which requires two fluorescent proteins, into a single wavelength indicator with a new color.

In a previous study, dual imaging of cAMP and Ca^2+^ was reported using CFP/YFP and a red fluorescent probe Fura Red [15]. Here, for further addition of a cGMP biosensor based on FRET, we engineered a blue fluorescent cGMP sensor using a blue fluorescent donor and the dark fluorescent acceptor. It is suited for multiplexing with the FRET-based cAMP sensor using CFP/YFP and Fura Red. Thus, using this combination of the sensors, we achieved triple imaging of the cyclic nucleotides and Ca^2+^ in a single cell.

## Results and Discussion

**Figure 1:**
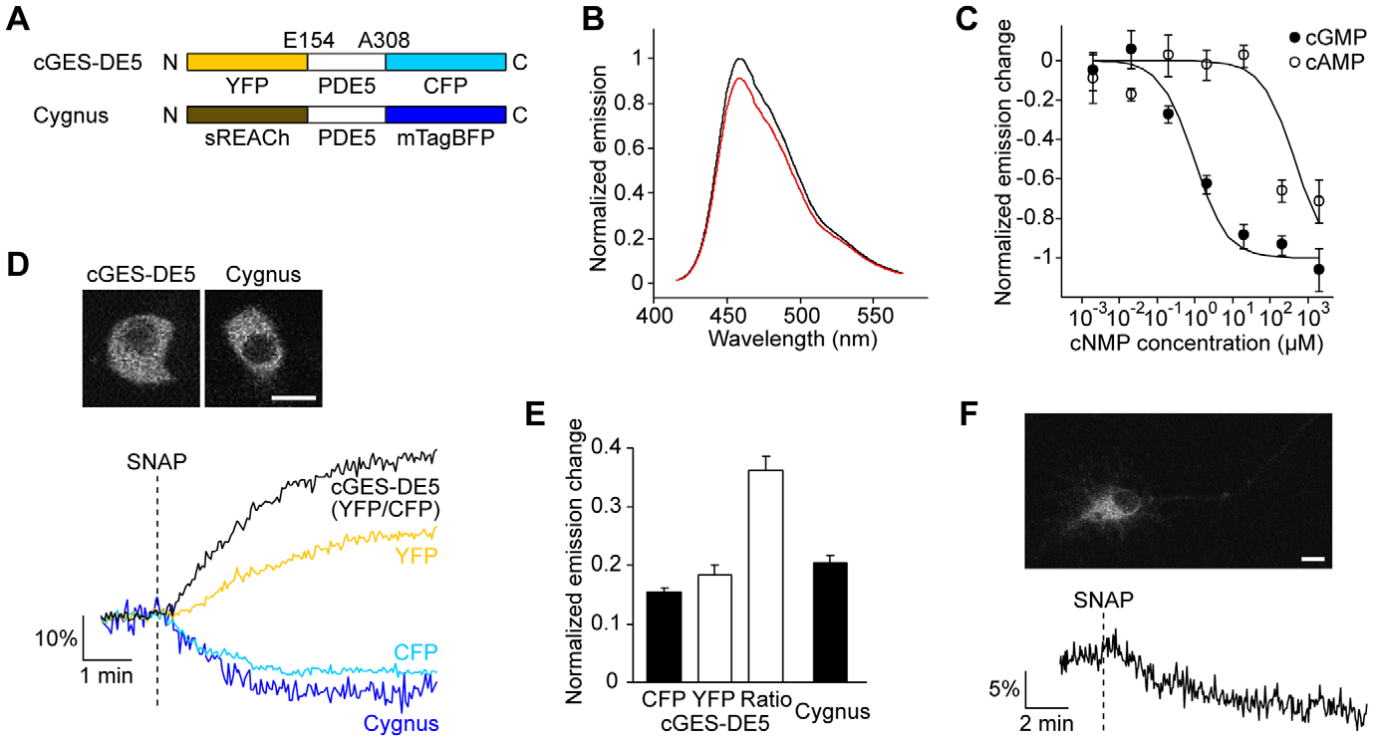
Blue fluorescent cGMP sensor Cygnus. (A) Domain structures of cGES-DE5 and Cygnus. (B) *In vitro* emission spectra of Cygnus at zero (black) and high cGMP (2 mM, red). (C) Concentration response curves of Cygnus for cGMP and cAMP. Half-maximal effective concentration (EC_50_) values for cGMP and cAMP were 1.0±0.2 µM and 0.4±0.3 mM (means ± s.e.m., *n* = 4), respectively. (D) Comparative measurements of cGMP dynamics in PC12 cells expressing cGES-DE5 and Cygnus. Representative fluorescence images (upper) and traces (lower) are shown. The cells were stimulated with 50 µM SNAP (*n* = 7). (E) Quantitative analysis of the maximal response amplitude in (D). Black and white bars indicate the decrease and the increase of the signals, respectively. (F) cGMP imaging in primary rat hippocampal neurons using Cygnus. A representative fluorescence image (upper) and a trace of the response to 50 µM SNAP (lower) are shown (*n* = 6). Scale bar, 10 µm.

A bright blue fluorescent protein mTagBFP was recently generated from the *Entacmaea quadricolor* RFP [16]. Its emission has a smaller spectral overlap with the absorption of YFP than that of CFP, but the estimated Förster radius (*R*_0_) of mTagBFP with YFP (4.9 nm) is comparable to of the CFP/YFP pair [17] due to the high quantum yield of mTagBFP (0.63). Therefore, we used an improved dark YFP sREACh [14] as a quenching acceptor for the blue fluorescent donor. Similarly to a previously reported cGMP sensor cGES-DE5 [18], we designed a biosensor, named Cygnus (for cGMP unicolor fluorescent sensor), with a cGMP binding domain from a phosphodiesterase (PDE5) sandwiched between mTagBFP and sREACh (Figure 1A). We investigated cGMP affinity and selectivity of the sensor using isolated proteins from transiently transfected HEK293T cells (Figure 1B and 1C). Upon addition of cGMP, Cygnus showed a decrease of the fluorescence (Figure 1B), which means an increase in FRET efficiency similar to cGES-DE5. Although the fluorescence signal change was small, this sensor had high cGMP affinity (1 µM) and selectivity for cGMP over cAMP (400-fold) comparable to cGES-DE5 (Figure 1C).

To examine the ability to monitor cGMP dynamics in living mammalian cells, we transfected either Cygnus or cGES-DE5 into rat pheochromocytoma PC12 cells, and compared the responses to the cGMP increase caused by a NO donor *S*-nitroso-*N*-acetylpenicillamine (SNAP) (Figure 1D and 1E). Cygnus exhibited a sufficient response with a decrease in the fluorescence as rapidly as cGES-DE5. The response amplitude was comparable to of CFP fluorescence in cGES-DE5, although it was small relative to of emission ratio of YFP/CFP. In HEK293T cells expressing Cygnus, we observed a transient response to SNAP (Figure S1). The recovery of the signal is consistent with a previous report that cGES-DE5 detects a degradation of the increased cGMP [18], confirming the reversibility of Cygnus. This sensor also reported the cGMP production in rat hippocampal neurons (Figure 1F), demonstrating the potential applicability of the sensor in the neurons relevant to the NO/cGMP pathway [19].

Finally, we tried triple-parameter fluorescence imaging for cAMP, cGMP and Ca^2+^ in a single cell. For combination with Cygnus, we attempted to use the FRET-based cAMP sensor with CFP/YFP Epac1-camps [20] for cAMP and the red fluorescent probe Fura Red for Ca^2+^. To confirm the spectral compatibility of these three sensors, we observed HeLa cells expressing either Epac1-camps or Cygnus and the untransfected cells loaded with Fura Red (Figure S2). Alteration of excitation wavelengths and detection with proper emission wavelength ranges permitted us to detect the signals individually from each of the sensors without significant spectral bleedthrough.

**Figure 2:**
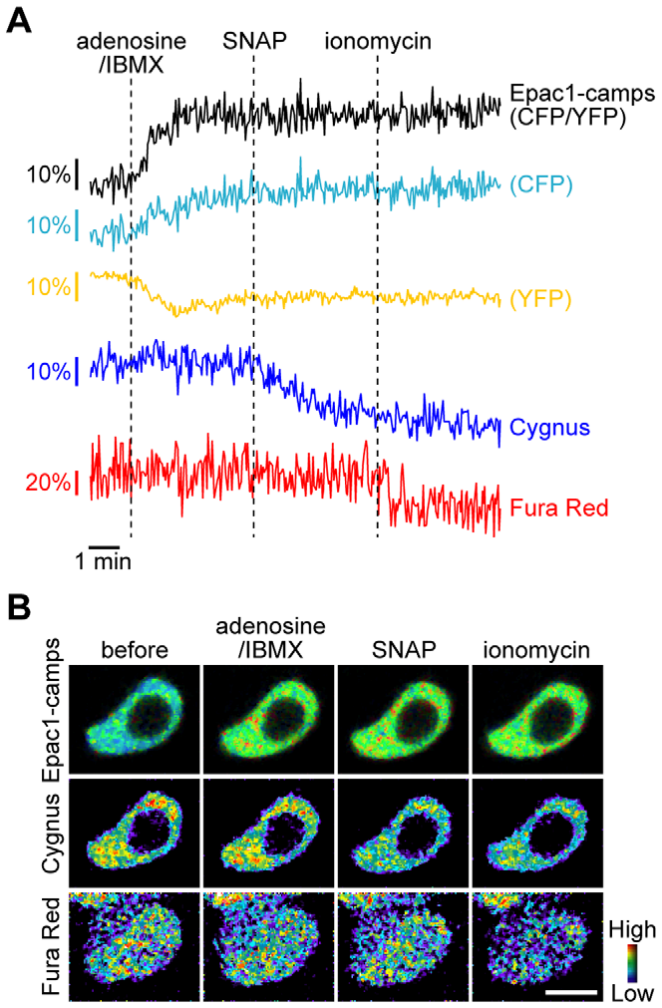
Triple-parameter fluorescence imaging of cAMP, cGMP and Ca^2+^ in PC12 cells. The cells coexpressing Epac1-camps and Cygnus and loaded with Fura Red were first stimulated with 5 µM adenosine and 100 µM IBMX, then with 10 µM SNAP and subsequently with 5 µM ionomycin (*n* = 6). Representative traces (A) and pseudocolored images before and at four minutes after the indicated stimulations (B). Fura Red was also loaded into the cells around the cell of interest. Scale bar, 10 µm.

To test the ability of this combination to monitor the dynamics of the three second messengers, we loaded Fura Red into PC12 cells coexpressing Epac1-camps and Cygnus (Figure 2). Since PC12 cells express endogenous adenosine A_2A_ receptors and the cAMP response to adenosine is known [21], we thus stimulated the cells first with adenosine and a phosphodiesterase inhibitor isobutylmethylxanthine (IBMX) to induce intracellular cAMP increase. This stimulation increased the emission ratio of CFP/YFP in Epac1-camps, whereas the fluorescence signals from Cygnus and Fura Red did not change. When subsequently stimulated with SNAP, the Cygnus fluorescence decreased but the signals from the other sensors were unchanged. After application of a calcium ionophore ionomycin, Fura Red detected the elevation of intracellular Ca^2+^ with a decrease in the fluorescence. These results indicated that this combination of the sensors is sufficient to individually monitor the dynamics of the cyclic nucleotides and Ca^2+^. And no response of Cygnus to the adenosine/IBMX stimulation, inducing cAMP increase detectable by Epac1-camps, was observed, confirming sufficient cGMP selectivity of Cygnus in living cells as well as *in vitro* (Figure 1C). In the multicolor imaging experiment, the fluorophores were excited at multiple wavelengths repeatedly. But no significant changes of the fluorescence signals from each sensor were found during the imaging experiments in the same conditions without stimulation, indicating that these sensors have sufficient photostability and no photoactivation and photoconversion occurred (Figure S3).

Under our experimental conditions, the spectral bleedthrough that led to the problem was not observed in the triple-parameter imaging (Figure 2). But, for example, in the case where one of the biosensors is used at a substantially higher concentration than the others, the bleedthrough would cause artifacts in the multiple imaging experiments. Adjusting the balance of intensities of the sensors should be important for the multiple imaging, and linear unmixing would be helpful in the case where sensor concentration cannot be controlled. The utility of Cygnus to detect the cellular cGMP response to external application of SNAP was sufficient in the multiple imaging (Figure 2), but its signal amplitude is relative small. To detect subtle changes in intracellular cGMP, the sensor with higher cGMP sensitivity such as red cGES-DE5 [10] would be useful.

A readout, which is not affected by environmental factors such as pH, extended the employability of the sensor. In the FRET pair for Cygnus, extremely high pH stability of mTagBFP fluorescence (p*K*_a_<3.0) was reported [16], but there is no report for the absorption of sREACh. We therefore examined its pH sensitivity (Figure S4). sREACh, which contains a mutation Q69M of a pH resistant YFP Citrine [22], showed high pH stability (p*K*_a_ = 4.5).

Taken together, we showed that the new sensor with blue fluorescence is suited for multiparameter fluorescence imaging [23], [24] and has a potential as a complementary alternative to the cpGFP-based sensor for wide applications because of its high pH stability. In addition, it would also be applied for FLIM, similarly to a recent report using RFP as a FLIM-FRET donor to combine with a FRET-based sensor using CFP/YFP [8]. Cygnus and the approach using FRET with the dark acceptor should be useful to investigate the complex interplay among multiple biological processes in a single cell.

## Methods

### Gene construction

mTagBFP cDNA was obtained from pTagBFP-N (Evrogen). To generate sREACh [14] and Citrine [22], EYFP gene in pEYFP-Actin (Clontech) was mutated using the QuikChange II site-directed mutagenesis kit (Stratagene). To construct Cygnus, a gene of human PDE5A1 (amino acids 154–308) with 5′ *Eco*RI and 3′ *Xba*I sites encoding dipeptides (Glu-Phe and Ser-Arg, respectively) was sandwiched between sREACh and mTagBFP genes using these restriction sites. For mammalian expression, Cygnus gene was subcloned into the *Bam*HI/*Not*I sites of pcDNA3.1(+) vector (Invitrogen) with a Kozak consensus sequence (CCACCATG) at the 5′ end. To investigate the pH dependence of the absorption, YFP genes were subcloned into the bacterial expression vector pQE30 (Qiagen) at the *Bam*HI/*Hind*III sites. Epac1-camps [20] and cGES-DE5 [18] in pcDNA3 were kindly provided by Dr. Martin J. Lohse.

### Protein expression, *in vitro* spectroscopy and pH titrations

Cygnus was transiently transfected into HEK293T cells using FuGENE 6 (Roche), and one or two days after transfection, cells were washed three times with chilled PBS, scraped from the plate, and resuspended in 5 mM Tris-HCl, 2 mM EDTA (pH 7.3). Following lysis by sonication (1 pulse) for 5 s on ice, cytosol was obtained by centrifugation at 100,000 g for 30 min at 4°C, and analyzed with cGMP and cAMP (Sigma). Concentration response curves were determined from the change in the fluorescence at 456 nm.

YFPs with an N-terminal polyhistidine tag were expressed in *E. coli* XL1-Blue strain (Stratagene). Cultures were grown overnight at 37°C, and pellets were lysed by sonication in a solution of 25 mM Tris-HCl (pH 8.0), 1 mM β-mercaptoethanol and a protease inhibitor cocktail for use with bacterial cells (Sigma). Protein purification was carried out using His GraviTrap (GE Healthcare). Purified proteins were dialyzed into PBS (pH 7.4) using a PD-10 column (GE Healthcare). pH titrations of the absorption were performed by using the buffers containing 125 mM KCl, 20 mM NaCl, 0.5 mM CaCl_2_, 0.5 mM MgCl_2_ and 25 mM of ethanolamine (pH 10.0), TAPS (pH 9.0, 8.5), HEPES (pH 8.0, 7.5, 7.0), MES (pH 6.5, 6.0, 5.5), or acetate (pH 5.0, 4.5, 4.0).

We measured the fluorescence with a spectrophotometer F-4500 (Hitachi) and the absorption with U-2001 (Hitachi) or SpectraMax Plus 384 (Molecular Devices). The mTagBFP emission spectrum taken from the website of Evrogen and the EYFP absorption spectrum were used to calculate the Förster radius as previously described [13].

### Cell culture

PC12 cells, HeLa cells and HEK293T cells were obtained from RIKEN Cell Bank. PC12 cells were grown in Dulbecco's modified Eagle's medium (DMEM, Invitrogen) supplemented with heat-inactivated 10% horse serum (HS, Sigma) and 5% fetal bovine serum (FBS, Invitrogen), 50 units/mL penicillin and 50 µg/mL streptomycin (Invitrogen). HeLa cells and HEK293T cells were grown in DMEM with 10% FBS, antibiotics and antimycotics (Invitrogen) and in DMEM with 10% FBS, 100 units/mL penicillin and 100 µg/mL streptomycin, respectively. Primary cultures of rat hippocampal neurons were prepared from Wister rat embryos at embryonic day 17–19 (E17-19, Charles River Japan) using a dissociation solution (Sumitomo Bakelite), and grown in Neurobasal medium (Invitrogen) supplemented with B27 (Invitrogen), 2 mM L-glutamine, 50–100 units/mL penicillin, 50–100 µg/mL streptomycin with or without 50 ng/mL nerve growth factor (NGF, Alomone). Cells were maintained at 37°C in a humidified atmosphere containing 5% CO_2_.

### Cell imaging

Cells were plated on a 35-mm glass bottom dish (Iwaki). Glasses were coated with poly-D-lysine (Sigma) for PC12 cells and hippocampal neurons, and with type I collagen (Nitta-Gelatin) for HEK293T cells. For transient transfections, we used Lipofectamine LTX (Invitrogen) for PC12 cells, HeLa cells and hippocampal neurons, and FuGENE 6 (Roche) for HEK293T cells. Plus reagent (Invitrogen) was added for PC12 cells and hippocampal neurons. Hippocampal neurons were transfected a week after plating, and observed 2–5 days after transfection. PC12 cells were imaged in Krebs-Ringer-HEPES (KRH) buffer [21], and the other cells were imaged in Hanks' balanced salt solution (HBSS, Invitrogen).

Imaging experiments were performed using a confocal laser scanning microscope (FluoView FV1000, Olympus). Cells were imaged on an inverted microscope (IX81, Olympus) with a 20× objective or a 40× oil immersion objective for HEK293T cells and a 60× oil immersion objective for the other cells, and were maintained at 37°C during the experiments.

For observation of cells expressing Cygnus, we used a 405 nm laser diode for excitation and the emission was detected at 440–480 nm. cGES-DE5 was excited at 440 nm and the emission was split using a 500 nm dichroic mirror and detected at 460–500 nm (CFP) and 520–560 nm (YFP).

For triple imaging of Cygnus, Epac1-camps and Fura Red, PC12 cells transiently transfected with cDNAs encoding Epac1-camps and Cygnus were incubated with 10 µM Fura Red-AM (Invitrogen) with Pluronic F-127 (Invitrogen) in the imaging buffer for 30 min at 37°C, and then washed twice with the buffer and further incubated for 15 min in the buffer or the cultured medium for hydrolysis of the acetoxymethyl ester form of the dye. Cygnus, Epac1-camps and Fura Red were excited at 405 nm, 440 nm and 488 nm, respectively. The emissions were detected at 420–450 nm (Cygnus), 490–500 nm (CFP of Epac1-camps), 535–565 nm (YFP of Epac1-camps), and 655–755 nm (Fura Red) with dichroic mirrors at 490 nm, 510 nm and 560 nm. A beam splitter (BS 20/80) was used for the dichroic excitation mirror. Samples were scanned on each line sequentially by the three lasers.

Timelapse interval was 2.5 s. Adenosine, IBMX, SNAP, and ionomycin were obtained from Sigma. Images were analyzed using MetaMorph software (Universal Imaging). In the analysis, images were subtracted background, and were smoothed with a 3×3 median filter to reduce noise for pseudocolored images of the triple imaging.

## Supporting Information

Figure S1 Intracellular cGMP monitoring in HEK293T cells using Cygnus. The cells were stimulated with 25 µM SNAP. A representative trace is shown (*n* = 6).
(0.17 MB TIF)

Figure S2 Spectral bleedthrough in the experimental conditions for triple-parameter imaging. (A) Representative fluorescence images in each detection channel of Cygnus-expressing, Epac1-camps-expressing and untransfected but Fura Red-loaded HeLa cells. Excitation (Ex) and emission wavelengths (Em) are as described in Methods. Scale bar, 10 µm. (B) Contributions of the signals of each sensor to the four channels (means ± s.e.m., *n* = 6).
(1.25 MB TIF)

Figure S3 Control experiments of triple-parameter imaging of cAMP, cGMP and Ca^2+^ in PC12 cells. Representative traces (A) and ratios of the signals in after to before experiments (B) (means ± s.e.m., *n* = 5). Signals of each sensor from Cygnus-expressing, Epac1-camps-expressing and untransfected but Fura Red-loaded cells were monitored as in Figure 2 without stimulation.
(0.25 MB TIF)

Figure S4 pH dependence of the absorption at 515 nm of EYFP, Citrine and sREACh.
(0.12 MB TIF)
