# Supplementary figures and images for: Blue Fluorescent cGMP Sensor for Multiparameter Fluorescence Imaging
Source: PLoS One. 2010 Feb 11;5(2):e9164. doi: 10.1371/journal.pone.0009164 (PMC2820094; doi:10.1371/journal.pone.0009164)

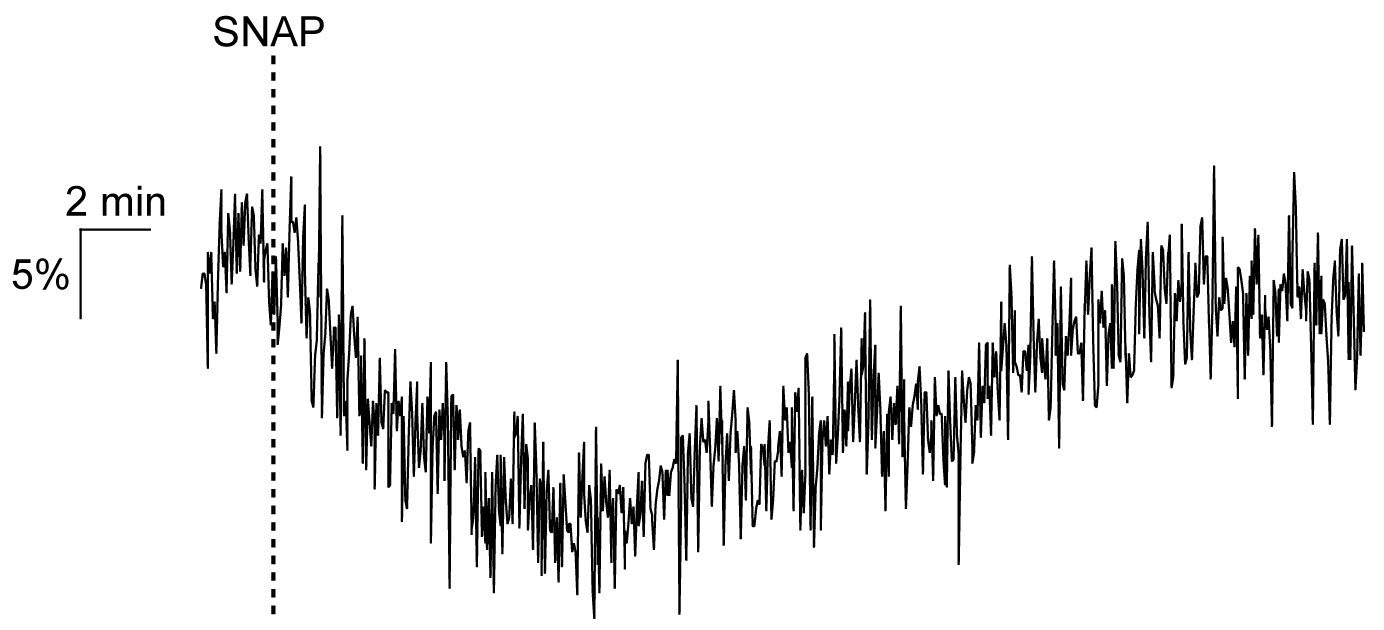

Supplement: Figure S1 — Intracellular cGMP monitoring in HEK293T cells using Cygnus. The cells were stimulated with 25 µM SNAP. A representative trace is shown (n = 6). (0.17 MB TIF) [file pone.0009164.s001.tif]

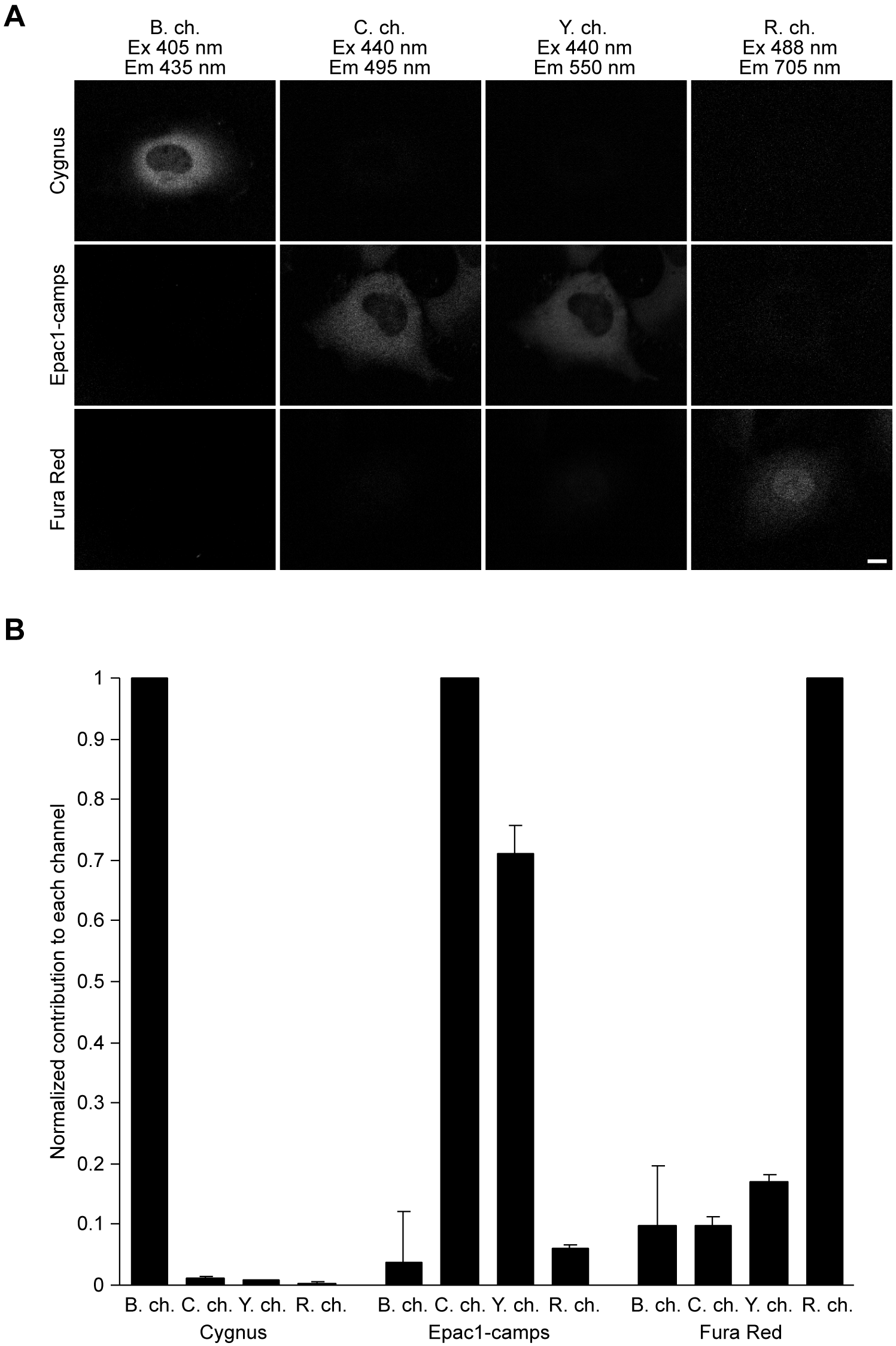

Supplement: Figure S2 — Spectral bleedthrough in the experimental conditions for triple-parameter imaging. (A) Representative fluorescence images in each detection channel of Cygnus-expressing, Epac1-camps-expressing and untransfected but Fura Red-loaded HeLa cells. Excitation (Ex) and emission wavelengths (Em) are as described in Methods. Scale bar, 10 µm. (B) Contributions of the signals of each sensor to the four channels (means ± s.e.m., n = 6). (1.25 MB TIF) [file pone.0009164.s002.tif]

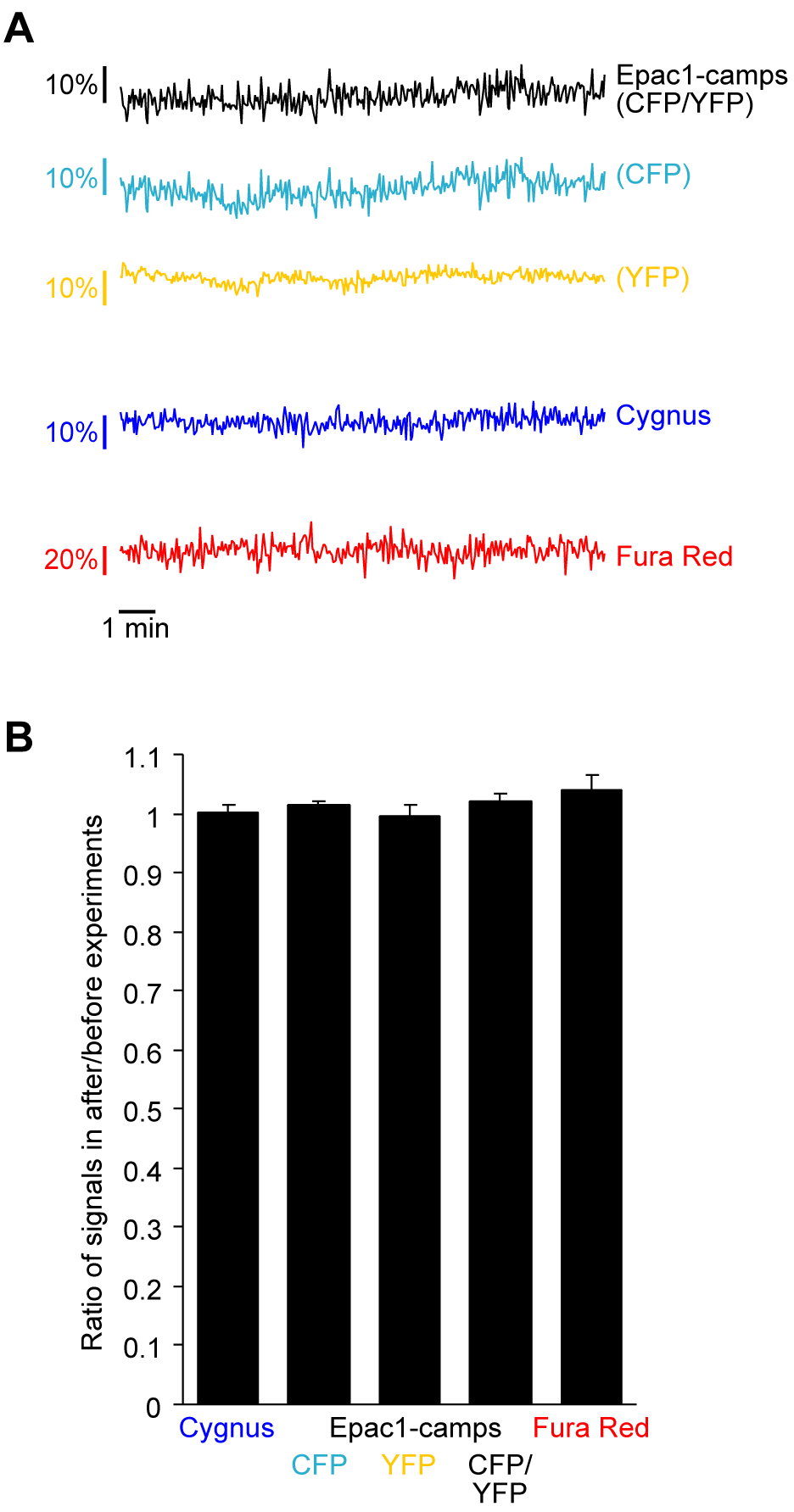

Supplement: Figure S3 — Control experiments of triple-parameter imaging of cAMP, cGMP and Ca2+ in PC12 cells. Representative traces (A) and ratios of the signals in after to before experiments (B) (means ± s.e.m., n = 5). Signals of each sensor from Cygnus-expressing, Epac1-camps-expressing and untransfected but Fura Red-loaded cells were monitored as in Figure 2 without stimulation. (0.25 MB TIF) [file pone.0009164.s003.tif]

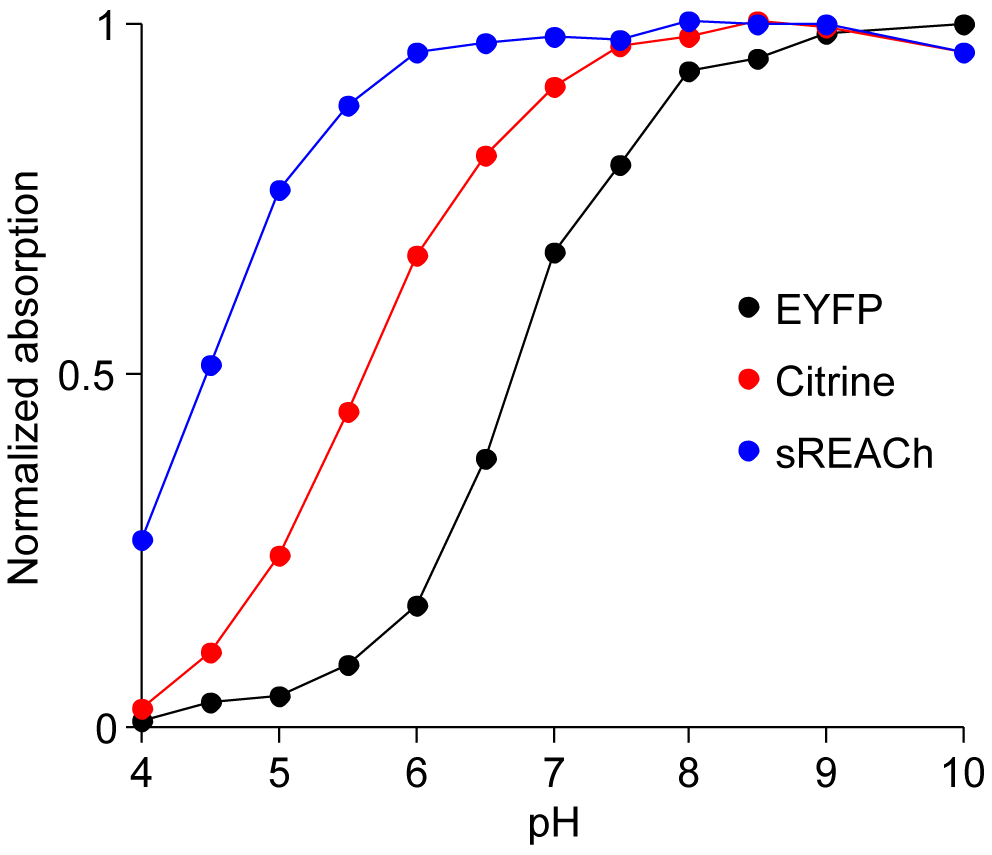

Supplement: Figure S4 — pH dependence of the absorption at 515 nm of EYFP, Citrine and sREACh. (0.12 MB TIF) [file pone.0009164.s004.tif]
